# Step by Step Towards Effective Human Activity Recognition: A Balance between Energy Consumption and Latency in Health and Wellbeing Applications

**DOI:** 10.3390/s19235206

**Published:** 2019-11-27

**Authors:** Enida Cero Dinarević, Jasmina Baraković Husić, Sabina Baraković

**Affiliations:** 1 Department for Information Technology, American University in Bosnia and Herzegovina, 75000 Tuzla, Bosnia and Herzegovina; 2 Department of Telecommunications, Faculty of Electrical Engineering, University of Sarajevo, 71000 Sarajevo, Bosnia and Herzegovina; jbarakovic@etf.unsa.ba; 3 Department for IT and Telecommunication Systems, Ministry of Security of Bosnia and Herzegovina, 71000 Sarajevo, Bosnia and Herzegovina; barakovic.sabina@gmail.com

**Keywords:** human activity recognition, health and wellbeing, HAR stages, energy consumption, latency

## Abstract

Human activity recognition (HAR) is a classification process that is used for recognizing human motions. A comprehensive review of currently considered approaches in each stage of HAR, as well as the influence of each HAR stage on energy consumption and latency is presented in this paper. It highlights various methods for the optimization of energy consumption and latency in each stage of HAR that has been used in literature and was analyzed in order to provide direction for the implementation of HAR in health and wellbeing applications. This paper analyses if and how each stage of the HAR process affects energy consumption and latency. It shows that data collection and filtering and data segmentation and classification stand out as key stages in achieving a balance between energy consumption and latency. Since latency is only critical for real-time HAR applications, the energy consumption of sensors and devices stands out as a key challenge for HAR implementation in health and wellbeing applications. Most of the approaches in overcoming challenges related to HAR implementation take place in the data collection, filtering and classification stages, while the data segmentation stage needs further exploration. Finally, this paper recommends a balance between energy consumption and latency for HAR in health and wellbeing applications, which takes into account the context and health of the target population.

## 1. Introduction

Human Activity Recognition (HAR) is defined as a classification process utilized for human motion recognition. HAR can be used in a broad range of applications, particularly health and wellbeing [1,2]. Creating a healthy lifestyle that includes regular physical activity, can be supported by collecting, assessing, and examining HAR data. Deficient physical activity, on the other hand, is one of the factors that precipitates a higher risk of stress occurrence, heart disease, diabetes, and repetitive motion injuries [3].

Various chronic diseases can be discovered and prevented using HAR [4]. Although the implementation of effective HAR applications is quite a hard and complex task as it allows the response for a specific patient, such as is the case with obese patients, diabetics, or heart disease patients [5]. Activity monitoring can be helpful in identifying abnormal activities and preventing unwanted outcomes related to dementia and other mental diseases [5]. Additionally, HAR can potentially be used in mental health applications because it can detect sedentary behavior, which is related to depression [6]. The monitoring of daily activities of older adults can help to identify long periods of inactiveness or the occurrence of a fall [3,7]. The aim of HAR in health applications is to enable interaction through monitoring/management between patients and medical staff (remote or in hospital). Besides this role, HAR also plays a key role with regard to assistance with daily activities aimed at preventing disease and preserving human health through the monitoring of daily sports activities, performance during sports activities and improvement in sports education enhancing human wellbeing. This paper discusses the overall HAR implementation in clinical health applications and applications for disease prevention and the preservation of health and wellbeing.

The implementation of HAR in the health and wellbeing domains carries specific requests in terms of energy consumption, recognition accuracy, and latency [8]. The energy consumption of sensors is challenging due to the high-energy requirements related to continuous monitoring of health conditions. The recognition accuracy is associated with the number of correct predictions of a classification model [9]. High recognition accuracy is crucial for the long-term monitoring of patients and for the promotion of technology adoption among operators, professionals and users [8]. Latency appears to be the main concern in HAR applications [7], since delayed activity recognition can have serious consequences in certain health and wellbeing applications (e.g., after a stroke or when sugar levels in blood decrease).

Inspired by all of the aforementioned, the aim of this paper was threefold: (1) to give an overall description of HAR through its stages, highlighting the influence of each stage on energy consumption and latency; (2) to review existing approaches for an energy-efficient and latency-sensitive HAR application and (3) to perform the grouping of current solutions based on context and health condition, emphasizing the prioritization of energy consumption and latency with regard to recognition of each group.

The remaining part of this paper is structured as follows: Section 2 describes the methodology used to conduct our research. Section 3 represents a literature review of approaches used to implement each stage of the HAR process. This section highlights the impact of each stage on energy consumption and latency. A summarization of current approaches for energy saving and latency minimization per stage during HAR in health and wellbeing applications is given in Section 4. This allows us to identify the research area for future work and gives valuable directions for the implementation of HAR in health and wellbeing applications. Finally, Section 5 entails the conclusion.

## 2. Research Methodology

The main objectives of this paper can be summarized as follows: (1) to provide a review of approaches used in each stage of HAR, emphasizing the impact of each stage on energy consumption and latency; (2) to identify approaches used to optimize energy consumption and latency by each stage of HAR; (3) to group the existing solutions in order to meet energy consumption and latency requirements, while considering the context and health condition of the target population. The research questions posed in this study were: (1) how do HAR stages affect energy consumption and latency? (2) Which approaches are used to minimize energy consumption and latency in current literature? (3) Can energy consumption and latency be managed at a specific HAR stage? (4) What other factors affect effective HAR implementation in health and wellbeing applications?

**Figure 1:**
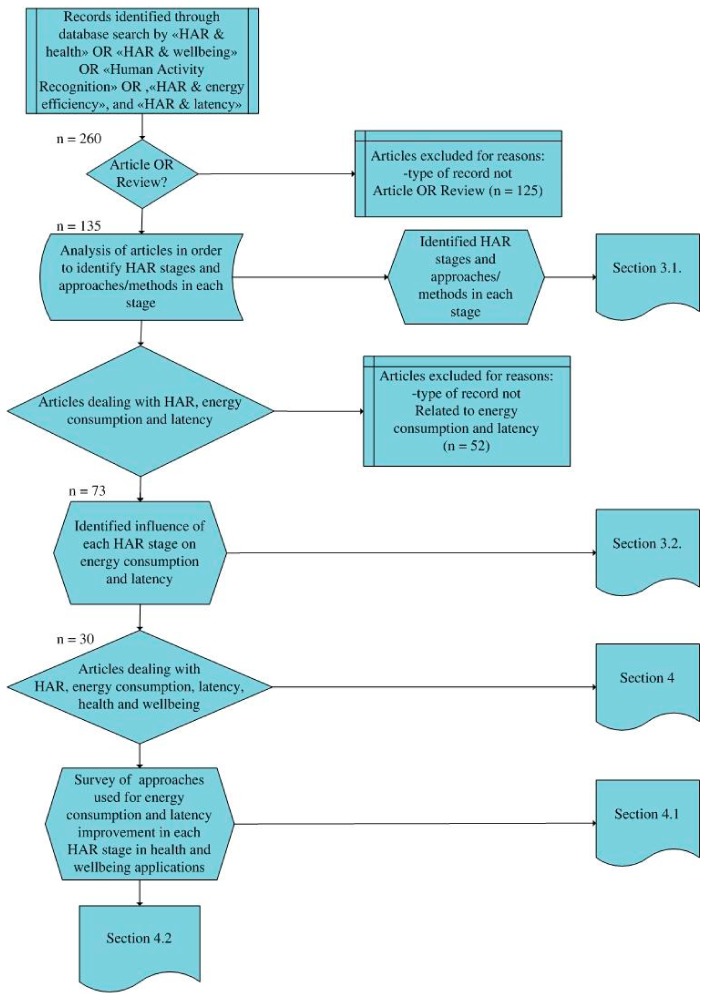
Research methodology.

The methodology used in the research is depicted in Figure 1. The first step was article collection, which resulted in the initial selection of 260 articles. The collection stage included a literature search that was carried out using a combination of the following keywords: activity recognition, HAR, HAR stages, health and wellbeing applications, energy consumption, and latency. The search was carried out using Google Scholar and Web of Science. Only 135 out of 260 collected articles were relevant for this research study (articles or reviews), while 73 articles were directly related to the HAR stages, energy consumption and latency. Out of 73 articles, 30 were related to HAR, energy consumption, latency and health and wellbeing applications (Figure 1). The articles were collected according to the year range from 2005 to 2019, however, most of them were published in 2018.

After the collection stage, the types of data which were not articles or reviews were excluded from the database. The next step was HAR stage identification and for each stage a summary of approaches were selected (Section 3.1). Additional paper selection was conducted in order to extract articles that dealt with energy consumption and latency in HAR. After the selection of articles, the authors explored the influence of the HAR stages on energy consumption and latency (Section 3.2). The next stage included the segregation of health and wellbeing applications where HAR was implemented across the literature collected, as well as the identification of approaches for energy consumption and latency reduction (Section 4). The next step was the survey of possible improvements for energy consumption and latency of HAR applications (Section 4.1) and the proposal of overall directions for the effective implementation of HAR in health and wellbeing applications (Section 4.2).

The investigation of different stages for HAR underlined the extent to which various approaches were covered by existing literature and also provided the basis for the identification of the influence of each HAR stage on energy consumption and latency. Based on the identified influence, the possibility to manage energy consumption and latency from different HAR stages was discussed. Having in mind an effective design for health and wellbeing applications, we proposed new grouping criteria, encompassing context and condition. Context is a three-dimensional variable that varies through the physical, user and medical states. The condition included different groups of health-related issues mentioned in the literature that were analyzed. Thus, we surveyed and compared different studies in this way in order to contribute to the understanding of the scope and grouping of HAR applications in health and wellbeing.

Our research methodology was motivated by the challenge of identifying how specific stages of HAR influence the energy consumption and latency of health and wellbeing applications. After the approaches in each HAR stage were identified, as well as their influence on energy consumption and latency, it became possible to choose approaches that best fit energy and latency expectations of HAR applications. In this sense, we proposed the grouping of health and wellbeing applications, the determination and prioritization of energy consumption and latency requirements of such application groups, thus leading to effective application implementation.

## 3. Impact of Human Activity Recognition (HAR) Stages on Energy Consumption and Latency

The challenges of HAR in health and wellbeing applications is mainly related to energy consumption and latency. Energy consumption is a crucial factor for certain applications of activity recognition, such as the long term monitoring of patients in health and wellbeing [8]. Furthermore, power efficiency along with computational efficiency appears to be the main challenge for wearable device-based HAR implementation [10]. Communications, sensing and computation tasks are generally the sources of energy consumption in HAR [8]. Continuous sensing and online updating of HAR data is required in HAR, notwithstanding that they are large consumers of energy [11]. However, research efforts in available literature for the reduction of energy consumption is limited.

Latency is defined as the time that has elapsed from the beginning of an activity to its detection by the system [8]. It encompasses the time required to acquire, process and analyze the data [8]. Low-latency classification is critical for certain HAR applications because immediate feedback may be required [12]. Sudden fall detection and epilepsy seizure detection are examples of such HAR applications in the health and wellbeing domain [8,13]. For some other applications, such as the distance walked in a day, latency can be less critical [8,12].

### 3.1. Overview of HAR Stages

**Figure 2:**
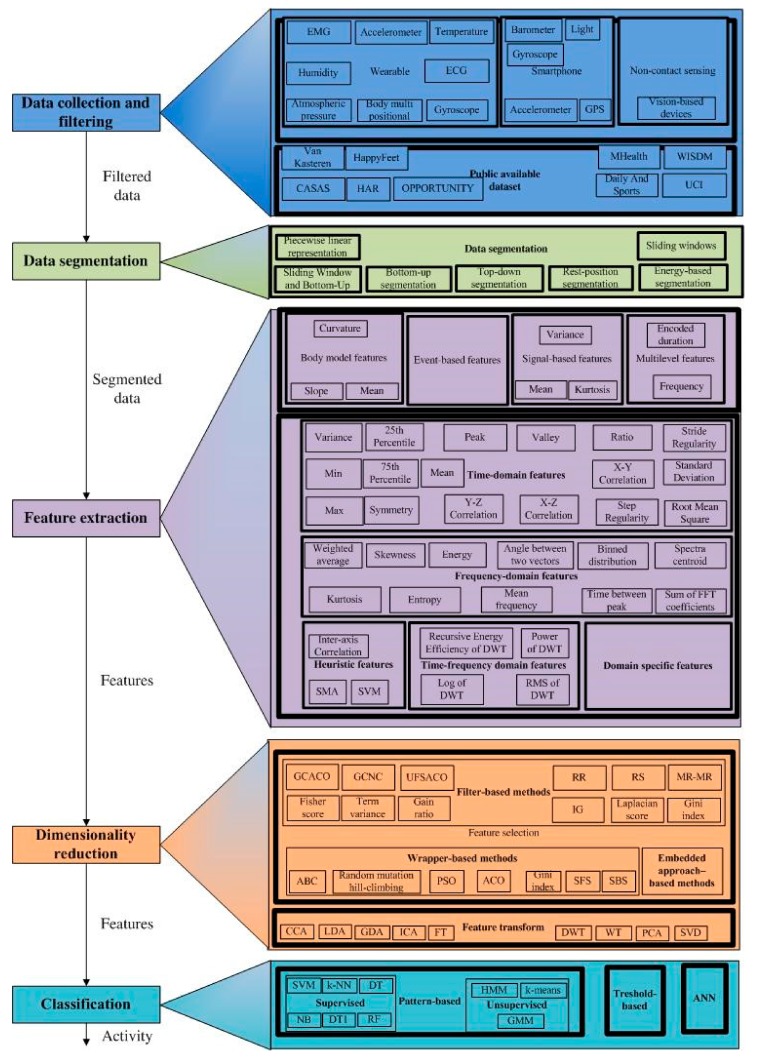
Overview of HAR approaches. Legend: Ant Colony Optimization (ACO), Artificial Bee Colony (ABC), Artificial Neural Network (ANN), Canonical Correlation Analysis (CCA), Decision Table (DT1), Decision Tree (DT), Discrete Wavelet Transform (DWT), Electrocardiogram (ECG), Electromyography (EMG), FFT (Fast Fourier Transformation), Gaussian Mixture Model (GMM), Generalized Discriminant Analysis (GDA), Global Positioning System (GPS), Graph Clustering based Ant Colony Optimization (GCACO), Graph Clustering with Node Centrality (GCNC), Hidden Markovian Model (HMM), Independent Component Analysis (ICA), Information Gain (IG), k Nearest Neighbors (kNN), Linear Discriminant Analysis (LDA), Naive Bayes (NB), Minimal Redundancy-Maximal Relevance (MR-MR), Particle Swarm Optimization (PSO), Principal Component Analysis (PCA), Random Forests (RF), Random Subspace (RS), Root Mean Square (RMS), Relevance Redundancy (RR), Sequential Backward Selection (SBS), Signal Magnitude Area (SMA), Signal Vector Magnitude (SVM), Singular Value Decomposition (SVD), Support Vector Machine (SVM), Unsupervised Feature Selection method based on Ant Colony Optimization (UFSACO), Sequential Forward Selection (SFS), Wavelet Transform (WT).

HAR is based on the recognition of daily human activities using various machine learning algorithms [14,15]. This is described through several stages, where different approaches can be used (see Figure 2). Some of the approaches for each stage of HAR that have been successfully implemented and have introduced motivation, as well as some examples of their use in HAR-related literature are mentioned in Section 3.1.

#### 3.1.1. Data Collection and Filtering

The first stage of HAR is data collection and filtering. The data collection process begins by defining a set of activities to be recognized, and then recording data from the sensors during a defined activity set [16], or simply taking over data from a publicly available HAR dataset [17].

Data capturing can be conducted by various wearable sensors, such as an accelerometer [3,14,18,19,20,21,22,23,24,25,26,27,28,29,30,31,32], a gyroscope [15,21,28,31,32,33,34,35,36], a magnetometer [19,34,35,36,37,38], an Electrocardiogram sensor (ECG) [31,39], a Global Positioning System (GPS) sensor [22], Electromyography sensor (EMG) [8,40,41], etc. Besides wearable sensors, data collection can be conducted using non-contact sensing [4,42,43,44], and with various sensors that are integrated into smartphones [45,46,47,48,49,50]. Many problems during data collection from wearable sensors may occur. For example, subjects forget to wear sensors, measurements have a high signal to noise ratio, bias in physical activity measurement from an accelerometer, and subjects that do not wear the device in an appropriate position [51]. On the other hand, wearable sensors enable the continuous measurement of physical activity (which is of particular importance to certain HAR applications in health and wellbeing) at a lower cost when compared to a non-contact sensing approach [52]. The disadvantage of non-contact sensing is the restricted area of measured physical activity (area with implemented sensors), and the high cost of implementation [52]. A particular benefit of non-contact sensors is the elimination of the possibility of forgetting to wear the sensors (important for people with mental disease) and the elimination of wear discomfort (important in some population groups such as people with skin disease) [52].

A vast number of datasets for HAR [39] are available for use (Table 1) such as: HAR [19,33,53,54], WISDM [53,55,56,57], UCI HAR [35,55,58], USCHAD [19], PAMAP2 [19,37,57], OPPORTUNITY [4,35,37], UniMiB-SHAR [4], MSR Action 3D [59], RGBD-HuDaAct [59], MSR Daily Activity 3D [59], MHEALTH [60], WHARF [22], KEH [61], etc. Determining which dataset to use in a HAR application and which techniques are the most appropriate for the HAR stages in a specific context is not a trivial task at all [39]. Since the performance level in activity recognition also depends on specific sets of activities [5], different authors have used various sets of possible activities in their research. After data collection, different preprocessing techniques are applied on raw signal data, in order to remove signal artefacts, such as noise and missing values [15,58]. If not removed, these artefacts badly decrease the classification algorithms performance [15]. Different preprocessing techniques can be found in literature. Filtering data with a third order low-pass Butterworth filter was used in [9] as a preprocessing technique. Besides filtering, certain authors used noise removal [62] and added additional data, such as time averaged signal magnitude of all accelerometer signals of three axis [62]. Preprocessed data must be segmented in order to be used in the subsequent stages of the HAR process.

#### 3.1.2. Data Segmentation

After preprocessing, the collected data is entered into the data segmentation stage, which is defined as the process of segment labeling, where each segment contains information about activities that have to be recognized [4]. In this stage, parts of information which are insignificant for recognition are removed [4]. Thus, the quantity of data is reduced. This is very important, since in each step a limited amount of data can be processed because of hardware-related constraints [4]. Piecewise Linear Representation (PLR) is simple for usage, since different data segments are linearly interpolated [63]. Simplicity and intuitiveness made sliding window algorithms popular in medical applications [28], leaving inconsistency in choosing a preferable window size [46]. Besides different sliding window sizes [64], some researches can also choose to introduce window overlapping [65]. The most commonly used fixed-size sliding window overlapping size is 1 s (used in [17,21,42,66]).

Besides the PLR and sliding window approaches, authors in [62] mention energy-based segmentation. This segmentation approach relies on the fact that various activities are present with various strengths, for a large number of activity recognition problems. Other segmentation approaches include rest-position segmentation [62], top-down segmentation [62], bottom-up segmentation [28,62], and Sliding Window and Bottom-Up (SWAB) [62]. After segmentation, data is prepared for feature extraction [62]. In certain studies, such as [67], segmentation is viewed as part of preprocessing.

#### 3.1.3. Feature Extraction

Feature extraction derivates various and broad features that are distinguishing for activities [62]. Deep learning methods, such as Convolutional Neural Network (CNN), and Recurrent Neural Network (RNN) can be used for feature extraction [10]. These methods do not require expert knowledge [68]. Extracted features can be classified into four categories: signal-based features, body model features, event-based features (e.g., features that characterize renewed eye movement) and multilevel features (e.g., data is clustered and then statistics are calculated on a sliding window) [62]. These five classes of features are the most commonly used in literature: time-domain features, frequency-domain features, time-frequency domain features, heuristic features and domain specific features [28,62]. The features are usually extracted in the frequency domain and/or the time domain [36]. Table 2 shows the most frequently analyzed features in the literature.

The time-frequency domain features indicate benefits over other domain features since they carry both time and frequency domain information [69]. They appear to be appropriate for capturing time-varying and non-stationary signals, that can be used to describe emotional status [70]. The Discrete Wavelet Transform (DWT) and the Hilbert Huang spectrum (HHS) are showing signs of future success in this field [70]. Features obtained from an essential understanding of how a unique set of movements can form distinguishable sensor signals, are called heuristic features [71]. Feature extraction is followed by dimensionality reduction in order to decrease the computational complexity and latency of HAR.

#### 3.1.4. Dimensionality Reduction

Dimensionality reduction is used to decrease the feature vector dimension while providing accuracy of recognition [62]. Basically, two forms of dimensionality reduction are used: feature selection and feature transformation [62].

In feature selection a subset of features is chosen from the original feature set [83], and a new feature vector with fewer features is used for activity description [33,77,83,84]. This approach is used in wearable sensor systems with limited hardware resources for real time activity recognition [33]. Feature selection approaches improve the initial baseline efficiency of HAR [21]. In general, feature selection approaches are divided into filter-based, wrapper-based, and embedded approach-based methods [19,83,84]. Nevertheless, some authors also use terms such as basic features (statistics applied to raw sensor data) and graphical features (generated from graph representations) [50]. However, this type of classification is rarely used. When filter-based methods are applied, the feature selection process is dispart from the classification verification process, which makes them fast, but their drawback is that they require a threshold to stop the feature selection process [84]. Table 3 lists the authors who have reported and implemented different filter-based methods for feature selection.

If a classification scheme is a wrapper around which the whole feature selection is carried out, then the approach for selecting features is called wrapper-based [75]. The main drawbacks of this approach are poor generalization across different learning methods and computational complexity. However, they tend to provide higher accuracy when compared to filter-based approaches. Different feature selection approaches can be found in literature (Table 4). Embedded approach-based methods completely remove noise and irrelevant features with filter-based methods, and create an optimal feature set using the wrapper-based method [79]. In this way, the high efficiency of the filter model is combined with the high accuracy of the wrapper model [79].

Feature transformation exploits the fact that the transformation of data onto a feature space with a lower dimension [28] results in dimensionality reduction [90]. Feature transformation is recommended if multiple features together provide good discrimination of activities [28], while they provide poor performance for individual differentiation [28]. Different forms of feature transformation appear in the literature. Some authors have proposed an unsupervised dimensionality reduction method based on the Common PCA (CPCA) method [28], while some combined PCA with Independent Component Analysis (ICA) [28]. An overall review of feature transform approaches is shown in Table 4. After dimensionality reduction, the data was prepared for classification.

#### 3.1.5. Classification

The choice of classification algorithms is a very important factor for HAR performance [62,78]. Basic approaches used in the HAR classification stage include threshold-based methods, pattern-based methods and Artificial Neural Networks (ANN). If activities can be distinguished by various intensities then threshold-based methods can be widely used [28]. Pattern-based methods are classified as supervised and unsupervised learning techniques. The most known supervised learning techniques include k-Nearest Neighbors (kNN) [4,18,71,91], Decision Tree (DT) [26,87,92,93,94], Decision Table [11], Random Forests (RF) [7,83], Naive Bayes (NB) [15,79,83,87,95,96] and Support Vector Machine (SVM) [2,12,33,34,58,60,76,79,81,83,88,97,98,99,100]. Classification techniques have been summarized in Figure 2.

kNN is known to be simple, robust [64], and the best solution among all supervised classification algorithms [83]. kNN seems to be the least complex algorithm [101], and its performance for fall detection implementation appears to be adequate [91]. The performance of kNN are directly related to the quality of the feature set, where low-quality features result in lower performance [12]. Furthermore, overlapping clusters (classes) lead to the poor performance of kNN [83]. A simple decision Tree [6] is comparable to kNN in terms of performance and computational complexity [11]. DT appears to be an adequate choice for activity classification with a hierarchy [11].

Authors in [83] showed that RF algorithms provided the highest average accuracy compared with SVMs, NBs, and DT [94]. On the other hand, RF drawback is the need for huge amounts of labeled data for good performance achievement [83]. The NB classifier is popular due to its simplicity [83,84,87], ease of implementation [83], and effectiveness [84]. For human activity recognition, the NB approach shows similar accuracy levels when compared to other classification approaches. Some studies claim that the NB approach outperforms other classification approaches. Other studies show that classification accuracy obtained when using the NB approach is lower in comparison when SVM and DT approaches are used [83]. Even SVMs demonstrated the worst results for classification in [2]. However, it was still better than MLP [99], kNN [58], and even ANN [33], and in some studies such as [12,34] produced the best classification result.

The Hidden Markov Model (HMM) was introduced to classification with the aim of improving activity recognition accuracy [4], relying on its unique advantage—capturing the transition among different types of activities [11]. The HMM classifier gives the best results among all unsupervised classification algorithms [83]. The main drawback of Gaussian Mixture Model (GMM) is a request for too many empirical parameters, which decreases the possibility of its implementation in practice [109]. However, in some cases, such as with the recognition of static postures and non-temporal event patterns, it appears to have good classification performance [88].

Additionally, k-means clustering has poor performance in the case of overlapping clusters (classes) [83]. However, they are still used in practice because of their advantages such as small computational complexity, high efficiency for large datasets, and a high linearity of time complexity [1]. Neural network usage is limited in practice because of its high computational cost and the need for a large amount of training data [5].

On the other hand, a high tolerance of noisy data makes them appropriate for some classification problems [60]. In [85] the authors reported that SVM, NN and RF approaches worked best for activity recognition.

Since Convolution Neural Networks (CNN) combine feature extraction and classification in an end-to-end approach [37], they can perform classification in a very efficient way [55,89]. Recurrent Neural Networks (RNN), however, outperformed CNN for short duration activities. While in some cases ANNs had better performance than other techniques, in other cases they appeared to be less effective [33].

### 3.2. Energy Consumption and Latency per HAR Stage

Most HAR stages affect energy consumption, whereas latency is affected only by the data collection and filtering stage, data segmentation stage, and classification stage. There are many articles dealing with energy consumption, whereas only a few of them are devoted to latency analysis.

#### 3.2.1. Impact of HAR Stages on Energy Consumption

Each HAR stage has been analyzed in order to determine its impact on energy consumption as follows:

- Data collection and filtering stage: Firstly, in the data collection and filtering stage the set of used sensors affects energy consumption [62,110]. The reduction in the number of sensors can help improve the energy efficiency of the sensor device [61], whilst adding new sensor-type events can improve accuracy [54]. The number of sensors in this stage also affects the ability for complex activity detection, which is easier done with more than a single sensor unit [10]. In health and wellbeing applications, new sensor types (especially wearables) can be impractical for elderly people [52] because they are a source of discomfort for them. Therefore, the choice of the number of sensors is a very complex problem in HAR. Energy consumption cannot be reduced by a reduction in the number of sensors in the case of smartphone-based data collection, since the number of sensors is already limited. Furthermore, in the case of non-contact sensing, the number of sensors depends of their type and the covered HAR area. Having this in mind, some authors measured energy efficiency of HAR approaches with wearables [8,10,14,30,31,32]. Some authors analyzed the energy consumption of activity recognition of smartphones [45,111,112].
- Data segmentation stage: Segmentation approaches also affect energy consumption, which is calculated through the computational complexity of a segmentation algorithm. As highlighted in Section 3.2, PLR cannot be used as a universal segmentation approach because of high computational complexity (and consequently energy consumption) [63]. Many online Piecewise Linear Approximation (PLA) approaches have been noted in literature, and some of them are introduced to reduce energy consumption in WSNs (Wireless Sensor Networks) [32]. Even increasing the window size improved the recognition accuracy of various complex activities and had a smaller effect on simple activities in most cases [113]. Therefore, the choice of activities in HAR affects the choice of the segmentation approach, and consequently computational complexity and energy consumption in this stage.
- Feature extraction stage: The approaches and type of extracted features from each segment of data can potentially influence the computational load (energy consumption) and classification accuracy [78] of HAR. Therefore, the choice of feature extraction approaches influences the battery life [5] of sensor devices. Keeping in mind the type of extracted features, it is worth mentioning that time-domain features reduce complexity because they avoid the framing, windowing, filtering, Fourier transformation, liftering, etc. of data [29]. Following the aforementioned, they can be deployed in nodes with limited resources [29], which is the case of many practical applications of HAR [114]. However, they have shown to be prone to measurement and calibration errors [29], which lowers HAR accuracy. Frequency-domain features are less susceptible to signal quality variations [21] and have a more robust performance [2]. The lack of temporal descriptions [70] appears to be the main drawback of frequency-domain features. In conclusion, time-domain features consume less energy compared to frequency domain features [10]. Other techniques for energy reduction mentioned in literature include the usage of locally extracted features [115] and Fast Fourier Transform (FFT) based features [32].
- Feature selection stage: Generally, feature selection causes an increase in computational and memory demands because it changes the shape of objects into high dimensionality vectors. This stage affects energy consumption through computational complexity of the selected algorithm. For example, the dimensionality reduction done using PCA helps reduce overall energy consumption [116].
- Classification stage: Classification approaches affect energy consumption through computational complexity of selected classification algorithms. For example, the complexity of RF was higher than in SVM and NN classifiers, resulting in higher energy consumption [10].

Based on the aforementioned, one can notice that every HAR stage affects energy consumption. In addition, several other factors affecting energy consumption have been identified. One of these factors is the environment in which data needs to be collected. In controlled environments, energy consumption is not a challenge [19] while in real life different factors can influence energy consumption. Some of these factors include the use of different kinds of sensors in multiple devices [49], unevenly distributed datasets among various classes [12,55,117] and the wrong placement or orientation of wearables sensors [15,62]. A lower performance of HAR in real-life environments raises the criteria for better energy consumption.

#### 3.2.2. Impact of HAR Stages on Latency

The impact of each HAR stage on latency is analyzed as follows:

- Data collection and filtering stage: Preprocessing techniques (filtering) cause additional latency during HAR [3]. These techniques should be avoided for low latency real-time applications of HAR [3].
- Data segmentation stage: In this stage, latency can be reduced using advanced methods for data segmentation [39]. The choice of window size exhibits a high influence on latency during HAR. On the other hand, optimal size is not defined a priori [10]. Intuitively, by decreasing the window size, activity detection increases [98] and energy needs decrease [13]. However, short window usage has higher overheads because the recognition algorithm is triggered more frequently. In a popular segmentation technique, the sliding window technique, the window size of 1–2 s can be the best tradeoff between accuracy and recognition latency [10].
- Classification stage: Classification algorithms also affect latency during HAR. Long latency of HAR during the testing stage is achieved using the NN classifier, while RF, ANN, and SVM classifiers [80] show similar behavior.

Based on the aforementioned, it can be seen that not all HAR stages affect latency. Latency is mainly affected by techniques used in the data collection and filtering stage, data segmentation stage, and classification stage.

### 3.3. Summary

Section 3 describes approaches and techniques for identifying activities across all stages of the HAR process. This is very important in order to gain insight into the large number of approaches at each HAR stage that can be found in literature. Furthermore, it is important to highlight that different approaches/combinations result in different recognition accuracies. Given that accuracy above a certain threshold is acceptable, it is very important to observe the effects of stages/combinations on energy consumption and latency. Not all approaches in all stages affect the individual performance parameter equally, so choosing the optimal approach in all stages would also lead to an optimal result for given energy and latency requirements. The importance of energy consumption and latency varies throughout application domains, but also within an application domain. A detailed approach for selecting techniques/combinations for achieving optimal results for energy consumption and latency creates the possibility of treating individual applications in an application domain, which further leads to the personalization of a given service. The goal of a detailed perception of all the possible approaches is to gain further insight into choosing the best approach for given application requirements in terms of energy consumption and latency.

Energy consumption is affected by all the stages of the recognition process, while latency is affected only by the data collection and filtering stage, data segmentation stage and classification stage. Based on the foregoing, it is conclusive that these stages are also critical for the process in which the choice of techniques/approaches should strike a balance between energy consumption and latency. The impact of a particular technique on energy consumption or latency is discussed in Section 3.2. For example, the NN algorithm, due to it being less complex, requires less energy consumption than the RF algorithm; on the other hand, the NN algorithm has a longer latency in recognition than the RF algorithm.

Not all health-related applications are equally sensitive to energy consumption or latency. Since accuracy is implied nowadays, energy consumption and latency should be balanced. Many research papers are focused on developing mechanisms to lower energy consumption and latency in the various stages of the HAR process. Section 4 provides a brief overview of these mechanisms entailed in the collected literature.

## 4. The Optimization of Energy Consumption and Latency in HAR

Section 4 identifies currently implemented HAR solutions in the literature that has been summarized (Table 5). These solutions are related to the following applications, such as active and assisted living (AAL) [103,118], fall detection (FD) [7,10,26,91,119,120], automatic estimation of activity capability for rheumatic and musculoskeletal disease (RMD) [121], monitoring of elderly people [38,75] and ambulatory monitoring (AM) [7,80]. Solutions combine different HAR techniques in diverse HAR stages, depending on their research goal. The HAR stage in focus is highlighted in Table 5 along with associated HAR approaches.

Table 5 shows that the applications of HAR in health and wellbeing are diverse and use different approaches in each stage thereby making it hard to analyze in the context of energy consumption and latency requirements. Generally, HAR designers face challenges associated with balancing energy consumption, latency, and required accuracy [8], which are regarded as the main performance parameters of HAR in health and wellbeing applications.

After the identification of factors that affect energy consumption and latency at each stage of HAR (Section 3.2), possible solutions for their improvement on a stage level are given in Table 6. Based on a summarization of HAR applications in health and wellbeing (Table 5), certain general directions for the effective implementation of HAR in health and wellbeing applications were formed. Finally, we identified a research area for future work based on currently considered improvements of energy consumption and latency for HAR applications in the health and wellbeing domain.

### 4.1. Improvements of Energy Consumption and Latency in HAR

Table 6 summarizes possible improvements for energy consumption and latency of HAR applications in the health and wellbeing domain.

Energy consumption can be improved by reducing the number of sensors [61], reducing the amount of data on the sensor node [8,32], reducing the sampling rate [14,30,61,82,111,124,125], using a dynamically adjusted sampling rate [124] and Kinetic Energy Harvesting (KEH) supporting devices, as well as adaptive selection of sensors in real-time data acquisition [61] in the Data collection and filtering stage of HAR. The impact of some of these mechanisms is verified in practice and listed in Table 6.

There are also mechanisms for energy consumption reduction in the data segmentation stage. The only verified example in literature is the use of the Piecewise Linear Approximation (PLA) algorithm [32]. In the feature extraction stage, savings in power consumption can be achieved in several ways, the use of time-domain instead of frequency-domain features [10], using locally extracted features instead of globally [115], multi-user activity recognition [115], and the calculation of the Fast Fourier Transform (FFT)-based features on wireless sensor nodes [32].

In the classification stage, several mechanisms for reducing energy consumption are applied such as energy efficient RF [10], template-matching approach [31], variable step size [45], adaptive accelerometer-based activity recognition [111], and the choice of a classification algorithm [10,124].

Latency can be reduced in the data segmentation and classification stages. A smaller window size can reduce latency during activity recognition [98], while an adequate classification algorithm can also have an impact on latency [80]. In addition to this, literature suggests avoiding preprocessing techniques, and the use of advanced methods for the representation of features and segmentation, to avoid greater latency during the HAR process [39]. It should be noted that not all HAR applications need to balance energy consumption and latency. For some HAR applications in the health and wellbeing domain, latency is not really an issue (daily sport activities), because no action needs to be taken after certain detected events. For others latency is critical (fall detection) because the detection of immediate danger or a problem should cause a reaction.

In addition to the impact of HAR stages, energy consumption and latency change depending on environmental parameters and the application of HAR. In that sense, Section 4.2 proposes directions for effective HAR design keeping in mind the factors mentioned.

### 4.2. Proposal of an Effective Design of a HAR Application in Health and Wellbeing

Most of the approaches for energy consumption and latency improvements of HAR in health and wellbeing are conducted in the data collection and filtering stages and then in the classification stage. As can be seen in Section 3, the key stages for achieving a balance between energy consumption and recognition latency are data collection and filtering, data segmentation and classification stages. Additional approaches to improve performance in the data segmentation stage should be explored. Since the data collection stage is related to users and their environment (context), the need for the introduction of context as one of the parameters for performance analysis is obvious.

Table 7 shows an overall approach to HAR implementation in health and wellbeing highlighting the importance of context and condition for improving energy consumption and latency. This table can be useful in the HAR implementation process to prioritize energy consumption requirements (through physical and user context) and latency requirements (through medical context and health condition). Further on, based on requirements related to energy consumption and latency, it is possible to determine the approaches for the implementation of each HAR stage.

As mentioned in Section 2, context is a three-dimensional variable including physical, user and medical contexts. From the physical context point of view, Table 7 delves into activities which were performed indoors [126,127] or indoors and outdoors [128]. Indoor activities could be those conducted in a medical institution [127,129] or a smart home [130]. In the indoor area (rehabilitation center and smart house), different approaches (Section 3.1.1) for data collection can be applied, which pose different challenges in relation to energy consumption.

Since HAR in rehabilitation centers was applied for the supervision of the elderly, having in mind the resistance of older adults to the sensory devices and the use of the smartphones [131], as well as the need for lower energy consumption, HAR implementation using non-contact sensing should be explored in detail in this scenario. In addition, a non-contact sensing approach can reduce performance costs in rehabilitation centers compared to smart homes, as the system is implemented for a specific group of people. In such enclosed spaces, personalization of the solution is very important and the problem of integration of HAR with other systems in the center/home is encountered. In the case of HAR indoor/outdoor, it is necessary to research the energy performance of combined sensing approaches (wearables and non-contact sensing) for data collection. If energy consumption is controlled in the other stages of the HAR process, then some of the energy-efficient mechanisms can be implemented. User context can be analyzed in terms of older adults [83,126,127,129] or other populations. Older adults (often referred to as elderly in the analyzed literature) with regard to this study were people aged 65 years and over. This was adopted based on findings from the [132] study. Other populations were in accordance with those under the age of 65.

Medical context refers to the presence/absence of a reaction when a specific event is detected and the time in which activity data is collected, which further affects the time needed for data analysis. Regarding the medical context, three situations can arise. The first is activity management [133,134], the second, activity monitoring [7,83,126,128,129] and third, activity encouraging [135,136]. Activity management requires a reaction to a specific detected activity/condition, while activity monitoring and activity encouraging require only activity information collection. Activity encouraging is conducted at a specific period of time and in specific conditions and contexts (Table 7), while monitoring and management require time independent tracking of activities.

In health and wellbeing applications, under the term chronic disease, activity recognition is most often referred to with regard to cardiac disorders [128,137,138,139], diabetes [8,128,138,139,140,141], obesity [8,128,138] or arrhythmia [8,140]. A healthy population refers to population without any registered illness or disease. Accordingly, we proposed the prioritization of selected HAR performance requirements related to energy consumption and latency. Context and condition were further used to determine the energy and latency performance requirements. Energy is affected by the physical and user context, and latency by the medical context and health condition for HAR application.

Prioritization was conducted using three levels of priority: 1—Low, 2—Medium, 3—High. The priority for energy consumption in an indoor environment was 1, and in an outdoor environment 3. This was explained by the fact that in an indoor environment it is easier to reach the energy source. Furthermore, the priority for energy consumption in a medical institution is 3, and in a smart home, 2 (driven by the fact that in a medical institution critical health conditions are treated).

Lower energy consumption is less important to the rest of the population compared to older adults (3 for the older adults, 2 for the others).

Latency had a priority of 3 for the management of activities, 2 for the monitoring of activities, and 1 for encouraging activities. Furthermore, latency had a priority of 1 for an ill population and 2 for a healthy population. The average priority for each category was calculated as an arithmetic mean. Based on average priority denoted as performance importance in Table 6, the most demanding in terms of energy consumption were indoor/outdoor systems for older adults, (3) followed by indoor/outdoor systems for the rest of the population (2.5). The priority required for energy consumption during activities in medical institutions for older adults was 2.34. Other groups of activities (older adults in smart homes and other population in medical institutions) had a priority of 2 for energy consumption.

In terms of latency in activity recognition, the highest priority or the smallest delay should be in cases of activity management for chronic patients (3), followed by the monitoring of activities for people with chronic disease (2.5), and a small priority (2) for activity monitoring for the healthy population and activity encouraging. It is clear that management (which requires a response to detected events) requires smaller latency when compared to monitoring. Latency in response to a specific medical condition should also have a higher priority when compared to changes in healthy people’s medical condition.

Section 4.2 explains the approach recommended for the application of HAR in health and wellbeing. It consists of observing the context (physical, user, and medical) and health status of the target population. Based on the input values of the mentioned parameters, priority is given to energy consumption and recognition latency. This priority will determine the required balance between these two performance parameters and will continue to influence the choice of approach at each stage of the process. It should be noted that the most demanding indoor/outdoor systems are those that manage the health of the chronically ill older populations due to high energy consumption requirements and low latency.

### 4.3. Summary

Section 4 highlights the key challenges of HAR applications in the health and wellbeing domain. The challenges were extracted by observing HAR implementations in the health and wellbeing domain from available literature and contemplating whether and how each stage of the process affected challenges in terms of energy consumption and latency. We have shown that the stages of data collection and filtering, data segmentation and classification stand out as key to achieving balance. Most of the approaches for overcoming challenges in existing literature take place in the data collection and filtering and classification stages, while the data segmentation stage needs further exploration. In the end of this section, recommendations for a balance between energy consumption and latency for general HAR application in health and wellbeing taking into account the context and health of the target population were given. Managing activities of the chronically ill older population in indoor/outdoor systems presents the greatest challenge for implementing HAR in health and wellbeing.

## 5. Conclusions and Future Work

This paper provides a comprehensive survey of current approaches used in each stage of HAR, highlighting the influence of each stage on energy consumption and latency, which are regarded as critical for real time HAR implementation in health and wellbeing applications. Currently considered approaches for energy consumption and latency reduction per HAR stage were summarized and directions for effective HAR implementation were proposed. The concept of HAR processes were introduced through the presentation of approaches and methods in each process stage: data collection and filtering, data segmentation, feature extraction, dimension reduction and classification. As a result, we gained insight into a multitude of approaches across all stages of HAR.

We considered whether and how each stage of the process affected challenges in terms of energy consumption and latency. Here, the stages of data collection and filtering and data segmentation and classification stood out as key to achieving a balance needed for real-time HAR applications in health and wellbeing, and were stages from which energy consumption and latency could be managed. Based on the distinct capabilities of HAR in health and wellbeing, it can be concluded that most of the approaches in overcoming challenges take place in the data collection and filtering and classification stages, while the data segmentation stage needs further exploration. Finally, this paper recommends a balance between energy consumption and latency for general HAR application in the health and wellbeing domain, which takes into account the context and health of the target population. In this paper, the context and health of the target population were regarded as factors that affect effective HAR implementation in health and wellbeing. Indoor/outdoor systems for managing activities of the chronically ill older population were the biggest challenge for implementing HAR in health and wellbeing.

A broad research community could use the results from this paper for further research in this area. Besides energy consumption and latency, other important challenges per HAR stage can be analyzed and the data segmentation stage could be further explored in literature. The results of this paper can be verified in different HAR implementations in health and wellbeing applications (initially through simulations and after that in practice). Some solutions for energy consumption reduction are already presented in literature [8,10,14,24,30,111,123,125]. Although certain solutions that balance energy consumption and accuracy exist [112], so far we have not found any papers covering energy efficient and latency sensitive solutions. The health and wellbeing community may benefit overall from effective HAR implementation. For example, continuous monitoring of health conditions (currently limited to energy consumption on sensors) can result in a better diagnosis, since it provides detailed information about an individual’s health. Furthermore, reduced latency in HAR recognition can prevent fatal consequences of slow reaction on elderly falls at home. In addition, hardware manufacturers and software developers of HAR solutions can benefit from the results of this paper, since it has demystified issues related to implementation, testing and comparisons of different approaches in each stage of the HAR process.

## Figures and Tables

**Table 1:** HAR dataset characteristics.

Dataset	Number of Activities/Actions/Class of Activities	Number of Involved Users	References
HAR	6/0/0	30	[19,33,53,54]
WISDM	6/0/0	36	[53,55,56,57]
UCI HAR	6/0/0	30	[35,55,58]
USCHAD	12/0/0	14	[19]
PAMAP2	12/0/0	9	[19,37,57]
OPPORTUNITY	5/0/0	12	[4,35,37]
UniMiB-SHAR	0/0/17	17	[4]
MSR Action 3D	0/30/0	1	[59]
RGBD-HuDaAct	12/0/0	30	[59]
MSR Daily Activity 3D	15/0/0	10	[59]
MHEALTH	12/0/0	10	[60]
WHARF	5/0/0	17	[22]
KEH	9/0/0	8	[61]

**Table 2:** Review of the most frequently analyzed features.

Feature Domain	Measured Physical Signals	Feature Calculation	References
Time, Frequency, and Heuristic domain	Data from accelerometer	Min, Max, Mean, SD, SMA, SVM, Tilt angle, PSD, Signal entropy, Spectral energy	[2]
Time and Frequency domain	Data from 3-axis accelerometer	Mean, Min, SD, Variance, MED, Skewness, Kurtosis, Energy, Principal frequency, Magnitude of principal frequency (for each axis of a 3-axis accelerometer), Cross-correlation of accelerometer axis, MED crossing for each axis, 25th percentile for each axis, 75th percentile for each axis	[34]
Time and Frequency domain	Data from accelerometer	Mean, Skewness, Kurtosis, DFT, Autocorrelation	[35]
Time and Frequency domain	Data from 3-axis accelerometer, a 3-axis gyroscope and a 3-axis magnetometer	AMP, MED; MNVALUE, Max, Min, P2P, STD, RMS, S2E	[36]
Time and Frequency domain	Data from accelerometer, gyroscope and a magnetometer	Mean, STD, MED, Min, Max, Skewness, Kurtosis, Energy, Entropy, IQR	[38]
Time domain	Data from accelerometer, compass sensor, gyroscope and a barometer	Min, Max, Mean, SD	[46]
Time and Frequency domain	Data from 3-axial acceleration	Mean, Variance, SD, Min, Max, Range between min and max, Absolute Min, Coefficient of variation, Skewness, Kurtosis, 1st Quartile, 2nd Quartile, 3rd Quartile, IQR, MCR, Absolute Area, DFR, Energy, Entropy, TAA, TMA, Correlation Corr(X,Z) CorrXZ Corr(Y,Z)	[61]
Time, Frequency, and Heuristic domain	Data from acceleration	Mean, SD, RMS, Peak count, Peak amplitude, Spectral energy, Spectral power, SMA	[71]
Time and Frequency domain	Data from acceleration	Mean, SD, Absolute Max, First 3 peaks in power magnitude, Spectral entropy, Autoregressive coefficient, SMA	[72]
Time domain features	Data from acceleration, gyroscope, temperature, magnetometer and barometer	Mean, SD	[73]
Time and Frequency domain	Data from accelerometer	Mean, SD, IQR, RMS, Energy of FFT components, Entropy of FFT histogram	[74]
Time and Frequency domain	Data from 3-axial acceleration	Spectral energy, Spectral entropy, Mean, Variance, Mean Trend, WMD, Variance Trend, WVD, DFA coefficient, X-Z Energy uncorrelated (Spectral), Max, Difference acceleration	[75]
Time, Frequency, and Heuristic domain	Data from acceleration or gyroscope	Mean, SD, Max, Min, SMA, Average sum of the squares, IQR, Signal entropy, Autoregression coefficients, Correlation coefficient, Largest frequency component, Weighted average skewness, Kurtosis, Energy of a frequency interval, Angle between two vectors	[76]
Time and Frequency domain	Data from 3-axial acceleration	Min, Max, SD, Median, Mean, Skewness, Kurtosis, Absolute skewness, Absolute kurtosis	[77]
Time and Frequency domain	Data from accelerometer	Mean, SD, median, 25th percentile, 75th percentile, Peak, Valley, RMS, Principal frequency, Spectral energy, Entropy, the sum of FFT Coefficients grouped in four exponential bands	[78]
Time and frequency domain	Data from accelerometer	Mean, Variance, RMS, Mean absolute deviation, Range, Covariance, Quartile Deviation, Coefficient of correlation	[79]
Time and frequency domain	Data from wristband hand-dominated actions	Mean, Min, Max, Range of overall time, Variance, Kurtosis, Skewness, Cross-mean, Rate, Energy, Entropy, Percentage of energy each detailed wavelet components accounts for	[80]
Time and frequency domain	Data from 3D accelerometer, gyroscope, magnetometer, and ambient pressure sensor as well as linear acceleration, gravity, and orientation	Mean, Variance, SD, RMS, Mean crossing rate, Zero crossing rate, Skewness, Kurtosis, Entropy, Integration, SMA, Band power	[81]
Time and frequency domain	Data from 3-axial acceleration	Mean, SD, Median, 25th percentile, 75th percentile, Pairwise correlation, RMD, IQR, Mean crossing rate, Mean of movement intensity, Normalized SMA, Dominant frequency, Spectral energy, Spectral entropy	[82]

Legend: SD (Standard Deviation), SVM (Signal Vector Magnitude), SMA (Signal Magnitude Area), PSD (Power Spectral Density), FFT (Fast Fourier Transformation), DFT (Digital Fourier Transform), AMP (Amplitude of the signal), MED (Median of the signal), MNVALUE (Mean of the signal), Max (Maximum of the signal), Min (Minimum of the signal), P2P (Peak to Peak Amplitude) RMS (Root Mean Square Power) S2E (Stand to End Value), IQR (Interquartile range), MCR (Mean Crossing Rate), TAA (Total Absolute Area), TMA (Total Magnitude Area), WMD (Windowed Mean Difference), WVD (Windowed Variance Difference), DFR (Dominant Frequency Ratio), DFA (Detrended Fluctuation Analysis).

**Table 3:** Review of feature selection approaches.

Feature Selection Approach	Feature Selection Approach Type	References
Filter-based methods	MR-MR	[65,79]
	GCACO	[79]
	GCNC	[79]
	IG	[5,17,60,84,85,86,87]
	Gain ratio	[79,85,88]
	Term variance	[79]
	Gini index	[79]
	Laplacian score	[79]
	Fisher score	[79]
	RS	[79,89]
	RR	[79]
	UFSACO	[79]
Wrapper-based	SBS	[79]
	SFS	[46,79]
	ACO	[79]
	PSO	[79]
	GA	[2,39,79]
	Random mutation hill-climbing	[79]
	Simulated annealing	[79]
	ABC	[79]

Legend: Graph Clustering with Node Centrality (GCNC), Graph Clustering based Ant Colony Optimization (GCACO), Unsupervised Feature Selection Method based on Ant Colony Optimization (UFSACO), Genetic Algorithm (GA), Particle Swarm Optimization (PSO), Minimal Redundancy-Maximal Relevance (MR-MR), Information Gain (IG), Random Subspace (RS), Relevance Redundancy (RR), Sequential Backward Selection (SBS), Sequential Forward Selection (SFS), Ant Colony Optimization (ACO), Artificial Bee Colony (ABC).

**Table 4:** Review of feature transform approaches.

Feature Transform Approach	Feature Transform Approach Type	References
Feature transform	FT	[27,102]
	WT	[27,103,104]
	DWT	[27,104]
	LDA	[5,28,59,90]
	GDA	[90,105]
	CCA	[7,105,106,107]
	SVD	[4,108]
	PCA	[4,40,55,59,60,74]

Legend: Fourier Transform (FT), Wavelet Transform (WT), Discrete WT (DWT), Local Discriminant Analysis (LDA), Generalized Discriminant Analysis (GDA), Canonical Correlation Analysis (CCA), Singular Value Decomposition (SVD), Principal Components Analysis (PCA).

**Table 5:** Summary of HAR applications in health and wellbeing.

Applications	HAR Stage in Focus	HAR Approaches	References
FD	Classification	Two public databases, ANN, kNN, QSVM, EBT	[119]
FD	Data collection and filtering, Classification	Wrist-Worn Sensor, Feed-Forward NN, GA, SVM, DT, RBS	[26]
FD	Data collection and filtering	Kalman Filter, kNN	[91]
FD	Feature extraction, Classification	Temporal and Frequency features, LDA, CART, NB, SVM, RF, kNN, NN	[120]
FD	Feature extraction, Feature selection, Classification	Improved RF, PCF, HSW	[10]
FD, AM	Data collection and filtering, Data segmentation, Feature selection	RFID sensors, CCA, MLGL1, LSVM, kNN, RF, NB	[7]
Health and wellbeing monitoring	Feature extraction, Classification	Wearable sensors (accelerometers, gyroscope, and magnetometer), 1 s. window with no overlap, BT	[38]
AM	Data collection and filtering, Feature extraction, Classification	Wristband sensor, Statistics-, Frequency-, and Wavelet-domain features, NB, kNN, NN, SV, RF	[80]
AAL	Data collection and filtering	Radar Smart Sensor, DTFT	[103]
AAL	Classification	Smartphone sensors (accelerometer, gyroscope, and gravity sensor), C4.5 DT, NB, SVM, RF, BA, kNN, HMM	[118]
RMD	Data segmentation, Feature extraction and classification	Accelerometer, DTW, RR, LDA	[121]
Monitoring of elderly people	Data collection and filtering, Classification	Tri-axial accelerometer, Relief-F, kNN, NB	[75]

Legend: Fall Detection (FD), Ambulatory Monitoring (AM), Active and Assisted Living (AAL), Discrete-Time Fourier transform (DTFT), Genetic Algorithms (GA), Neural Network (NN), Support vector machines (SVM), Decision Trees (DT), C5.0 rule-based systems (RBS), k Nearest Neighbors (kNN), Artificial NN (ANN), Quadratic Support Vector Machine (QSVM), Pairwise Correlation Features (PCF), Hybrid Sliding Windows (HSW), Ensemble Bagged Tree (EBT), Rheumatic and Musculoskeletal Diseases (RMD), Dynamic Time Warping (DTW), Linear Discriminant Analysis (LDA), CART Decision Trees (CART), Gaussian Naïve Bayes (NB), Random Forest (RF), Hidden Markov models (HMM), Sequential Forward Floating Search (SFFS), Canonical Correlation Analysis (CCA), Multinomial Logistic Regression with 1 (MLGL1), SVM with linear kernel (LSVM), Local Energy based Shape Histogram (LESH), Sequential Minimal Optimization (SMO), Simple Logistic Regression (SLR), Bagged Trees (BT), Bootstrap Aggregating (BA).

**Table 6:** The summary of possible improvements of energy consumption and latency.

	HAR Stage	Improvement Approach	Verified in Literature
Energy consumption	Data collection and filtering	Reducing the number of sensors Reduce sensor data on the wearable sensor node Sampling rate reduction Dynamically appropriate sampling rates KEH Wearables Adaptive selection of sensors in real-time	[14,19,30,32,61,122,123]
	Data segmentation	PLA	[32]
	Feature extraction	Time domain-features instead of frequency-domain features Using locally extracted features for global multi-user activity recognition Calculation of FFT-based features on the wireless node sensor	[10,32,115]
	Classification	Energy efficient RF Template matching approach Variable step size Adaptive Accelerometer-based Activity Recognition controls the activity recognition duration The choice of algorithm for classification	[10,31,45,124]
Latency	Data segmentation	Decreasing the window size	[98]
	Classification	Choice of algorithm	[80]
	General	Avoid preprocessing techniques Advanced methods for the representation of features and segmentation	[39]

Legend: Human Activity Recognition (HAR), Kinetic Energy Harvesting (KEH), Piecewise Linear Approximation (PLA) of Fast Fourier Transform (FFT), Random Forests (RF).

**Table 7:** Input consideration for HAR implementation in health and wellbeing.

Context									Condition		Performance Importance		References
Physical				User		Medical					Energy	Latency	
Indoor	Outdoor	Medical Institution	Smart Home	Older Adults	Other Population	Activities Management	Activities Monitoring	Activities Encouraging	Chronic Disease	Healthy			
x		x		x		x			x		2.34	3	[30,129]
x			x	x			x			x	2	2	[7,83,103,126,130]
x			x	x			x		x		2	2.5	[140,142]
x		x			x			x	x		2	2	[127]
x	x	-		x			x			x	3	2	[128]
x	x				x		x		x		2.5	2.5	
